# Supplementary material for: Bismuth-Rich Co/Ni Bimetallic Metal–Organic Frameworks as Photocatalysts toward Efficient Removal of Organic Contaminants under Environmental Conditions
Source: Micromachines (Basel). 2023 Apr 22;14(5):899. doi: 10.3390/mi14050899 (PMC10222880; doi:10.3390/mi14050899)
Supplement: Supplementary file 1 [file micromachines-14-00899-s001.zip › micromachines-2336619-supplementary.pdf]

Supporting Information

Bismuth-Rich Co/Ni Bimetallic Metal–Organic Frameworks as Photocatalysts toward Efficient Removal of Organic Contaminants under Environmental Conditions

Ayesha Siddiqa 1, Toheed Akhter 1,*, Muhammad Faheem 1, Shumaila Razzaque 1, Asif Mahmood 2, Waheed Al-Masry 2, Sohail Nadeem 1, Sadaf Ul Hassan 3, Hyunseung Yang 4 and Chan Ho Park 5,*

1 Department of Chemistry, School of Science, University of Management and Technology, C-II, Johar Town, Lahore 54770, Pakistan

2 Department of Chemical Engineering, College of Engineering, King Saud University,

Riyadh 11451, Saudi Arabia

3 Department of Chemistry, COMSATS University Islamabad, Lahore Campus, Lahore 54000, Pakistan

4 Electronic Convergence Materials & Device Research Center, Korea Electronics Technology Institute, Seongnam-si 13509, Republic of Korea; hsyang@keti.re.kr

5 Department of Chemical and Biological Engineering, Gachon University, 1342 Seongnam-daero

* Correspondence: toheed.akhter@umt.edu.pk (T.A.); chhopark@gachon.ac.kr (C.H.P.)

Table S1: Calculations of crystallite size of Co/Ni-MOF and Co/Ni-MOF@BiOI composite using Scherer's equation.

**Co/Ni-MOF**			**Co/Ni-MOF@BiOI**		
**Peak Position (2θ)**	**FWHM (degree)**	**Crystallite size (nm)**	**Peak Position (2θ)**	**FWHM (degree)**	**Crystallite size (nm)**
11.85026	0.25406	31.43729138	26.11503	0.69764	11.09294
16.78405	0.36811	21.81487026	32.76448	0.38302	19.89912
21.77479	0.23122	34.98791176	33.32814	1.59936	4.758559
25.81226	0.2611	31.21480144	47.04654	0.61856	11.7759
28.2985	42.15449	0.194352757	54.71319	1.31534	5.364263
Average crystallite size	-	23.93 nm	Average crystallite size	-	10.58 nm

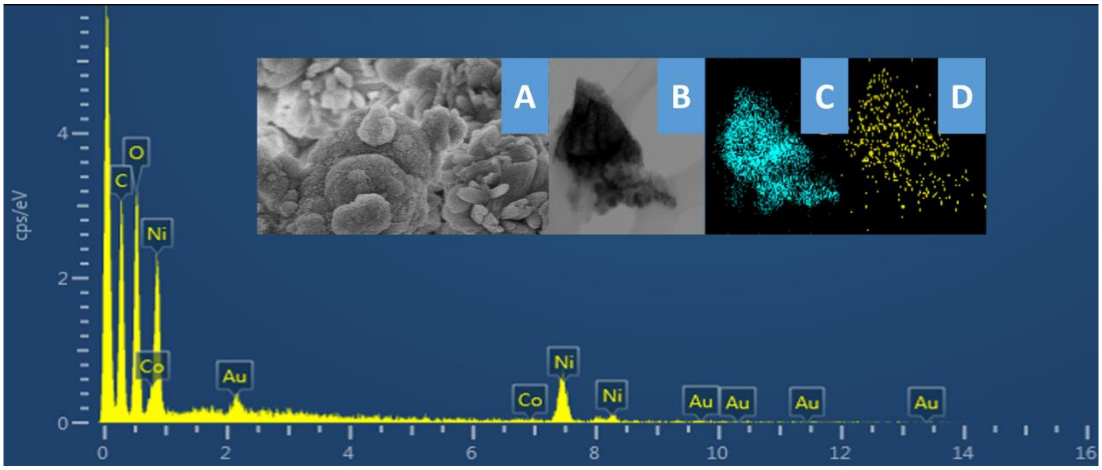

Figure S1. EDX spectrum of Co/Ni-MOF, (A) SEM of Co/Ni-MOF (500 nm resolution), (b) HR-TEM of Co/Ni-MOF, (C) Ni and (D) Co element maps in HR-TEM image.

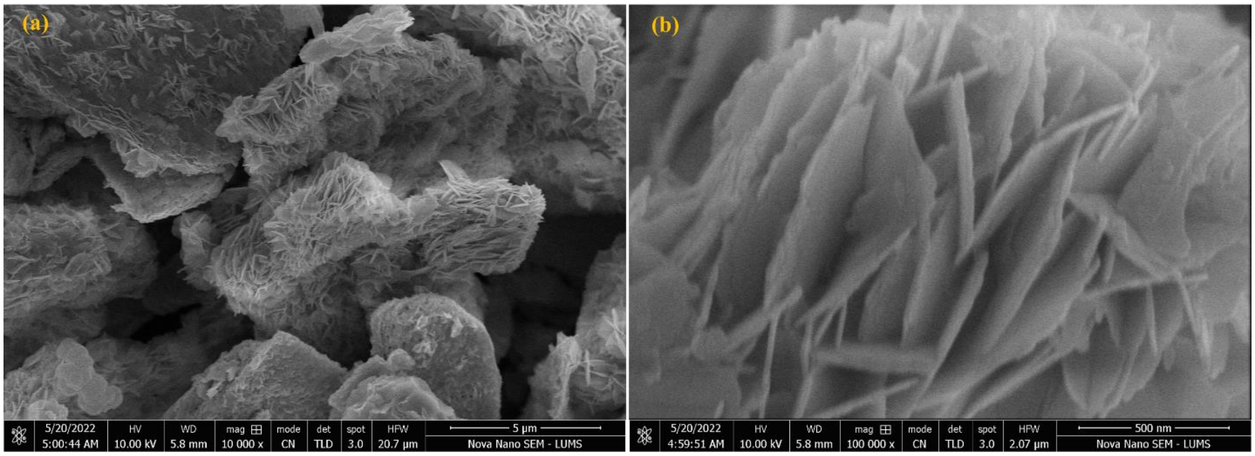

Figure S2 SEM micrographs of BiOI.

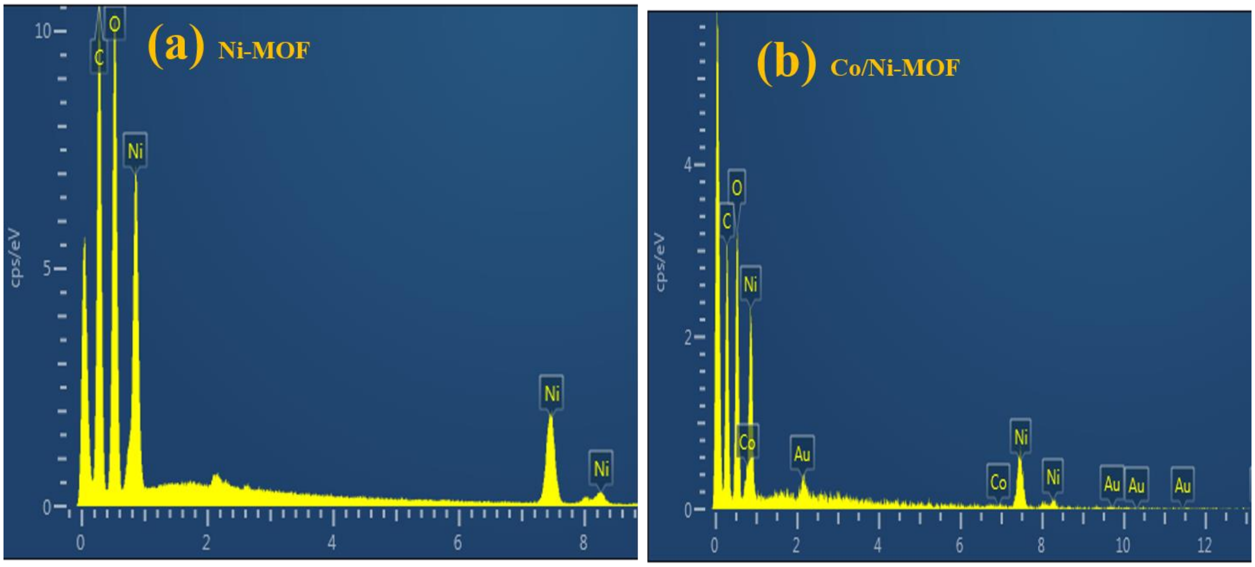

Figure S3 EDX spectra of (a) Ni-MOF, (b) Co/Ni-MOF

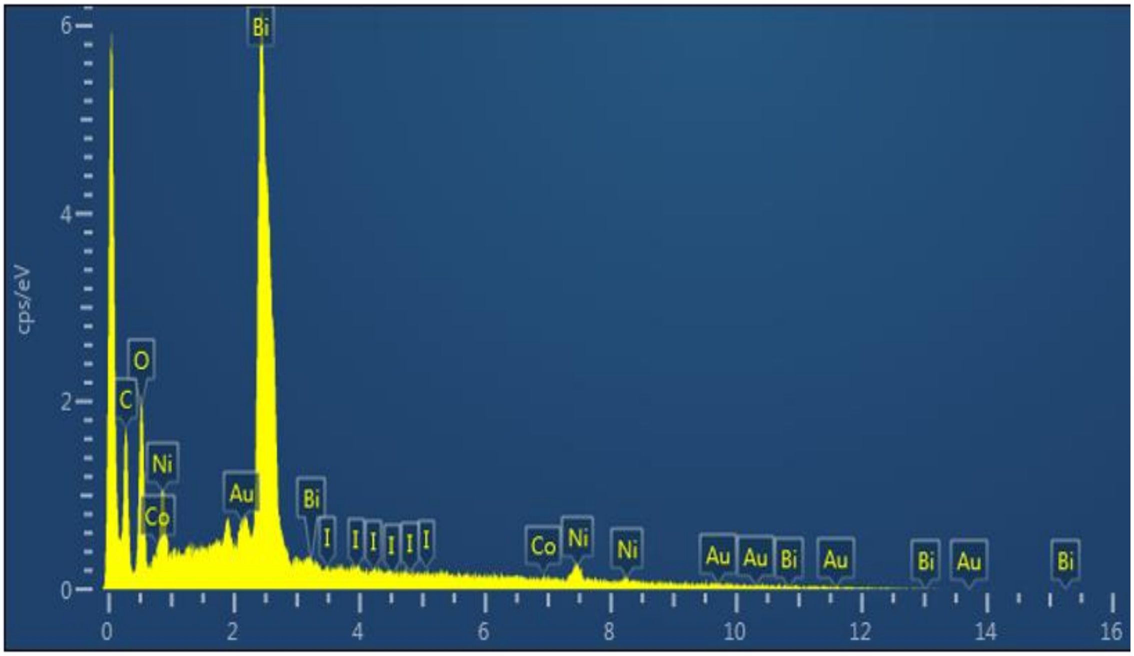

Figure S4. EDX spectra of Co/Ni-MOF@BiOI-10

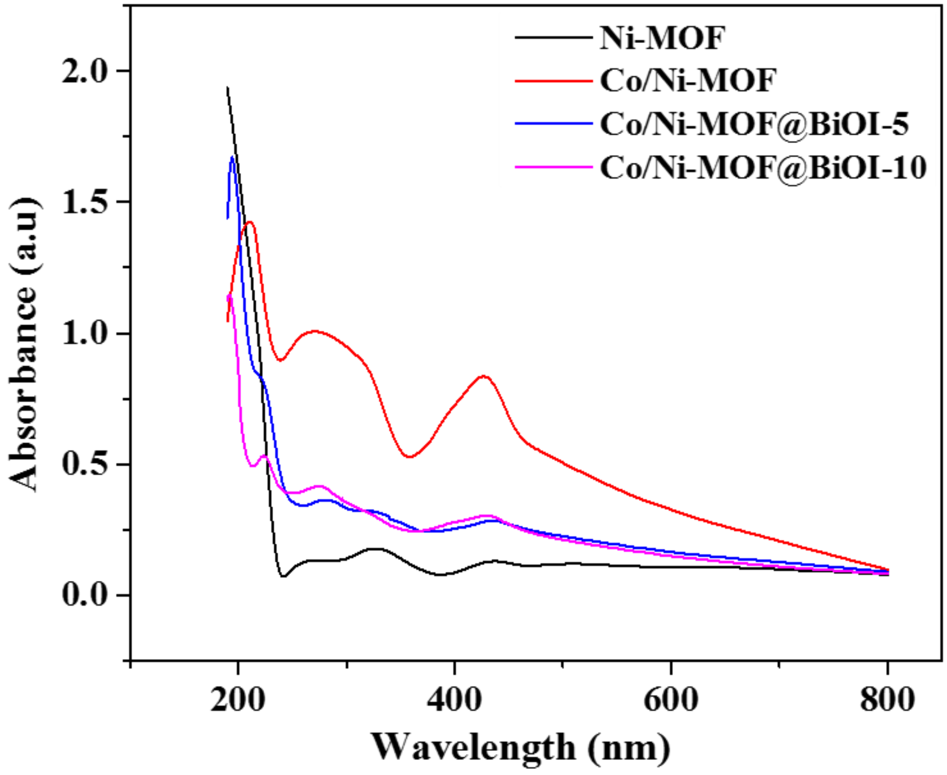

Figure S5. UV-vis spectra of all the samples.

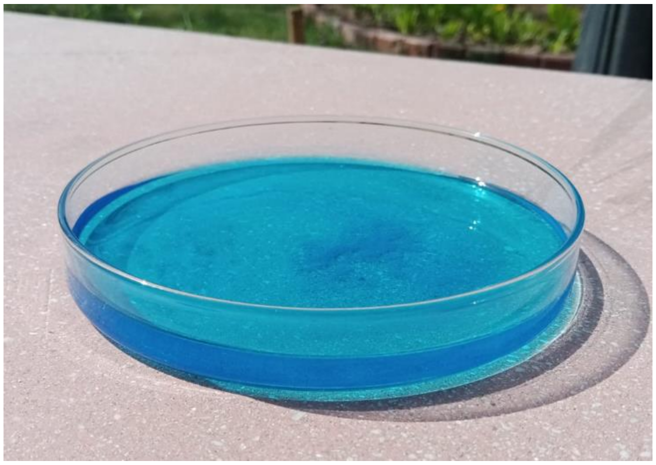

Figure S6. Irradiation of MB solution in sunlight. During irradiation, temperature was 35-38 °C, solar radiation intensity was 240 W/m², and relative humidity was about 35 %.

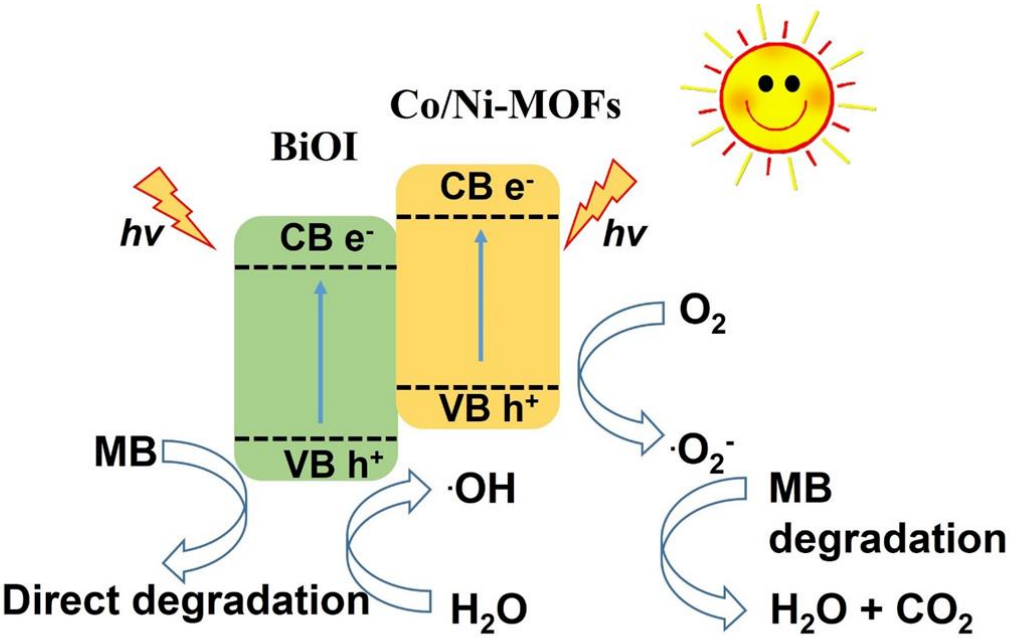

Figure S7. A schematic diagram of photocatalytic degradation of MB by Co/Ni-MOF@BiOI-10 under direct sunlight.
